# Human Contact Frequency as a Dominant Ecological Driver of Fungal Community Assembly and Homogenization in Public Built Environments

**DOI:** 10.4014/jmb.2602.02016

**Published:** 2026-04-03

**Authors:** Gui Nam Wee, Juchan Hwang, Boyeon Kwon, Jihyeon Kim, Seunghyun Kim, Sung Chul Seo, Soojin Jang

**Affiliations:** 1 Antibacterial Resistance Laboratory, Institut Pasteur Korea, Seongnam-si 13488, Republic of Korea; 2 The Environmental Health and Safety Institute, Seokyeong University, Seoul 02713, Republic of Korea; 3 Allergy Immunology Center, College of Medicine, Korea University, Seoul 02841, Republic of Korea; 4 Department of Nano, Chemical and Biological Engineering, College of Engineering, Seokyeong University, Seoul 02713, Republic of Korea

**Keywords:** Urban mycobiome, Fungal community, Indoor environments, Pathogen, Human impacts

## Abstract

As modern populations spend the majority of their time indoors, understanding indoor microbial ecology is crucial for public health. While research has addressed abiotic pollutants, the ecological dynamics of surface-associated mycobiomes remain insufficiently understood. This study assessed fungal communities across 25 types of public facilities in South Korea to evaluate the relative influence of environmental parameters and human-driven factors. A total of 327 surface samples from six surface types (handles, tables, chairs, walls, pillars, floors) were analyzed using internal transcribed spacer (ITS) sequencing, yielding 27 million reads and 31,721 amplicon sequence variants (ASVs). Although temperature and humidity significantly correlated with airborne fungal concentration, they exerted minimal influence on community diversity and structure. Instead, the intensity of human contact with indoor surfaces emerged as a primary driver of fungal community composition. We found that the relative abundance of the human-associated genus *Malassezia* is strongly associated with two distinct ecological states of indoor surface mycobiomes; high-*Malassezia* samples exhibited significantly distinct communities (ANOSIM R = 0.217, *p* < 0.001) and dense co-occurrence networks among genera of potential clinical relevance, with strong correlations between *Malassezia* and both *Aspergillus* and *Cladosporium* (|corr| = 0.81). These *Malassezia*-associated patterns persisting across diverse facilities demonstrate that human-driven microbes are the primary ecological drivers of surface mycobiomes in public spaces, providing foundational evidence for human contact-based microbial assessments in public health monitoring and hygiene-conscious environment design.

## Introduction

The indoor environment is the main living space where modern people spend most of their time, and it shares the space with numerous microorganisms that coexist and interact closely with human health [1]. Indoor microorganisms affect the development and aggravation of immune diseases such as allergies, asthma, and can also act as source for the transmission of opportunistic pathogens [2]. Therefore, understanding and managing indoor microbial communities is crucial in terms of public health and environmental management.

Previous studies have demonstrated that among various environmental factors such as ventilation and building materials, human presence and activity constitute the most dominant influences shaping indoor microbial communities [3]. Humans continuously release human-associated microorganisms from the skin and respiratory tract into the indoor environment and serve as vectors introducing microbes from the outdoor environment [4, 5]. However, existing studies have generally regarded humans as a single source of microbial input, and there is a relative lack of understanding regarding how specific human behaviors and surface functions interact to drive divergent microbial community assemblies. In addition, although fungi are major allergens and opportunistic pathogens, much of the existing research has predominantly focused on bacterial communities.

Indoor microbial research is typically conducted through air and surface sampling, each capturing distinct but complementary dimensions of the indoor microbiome. While air samples are useful for assessing acute respiratory exposure risk, surface samples are more effective for identifying the accumulated microbiological signatures formed through long-term microbial deposition and interaction. Importantly, airborne and surface microbial communities are linked through deposition and resuspension processes [6]: airborne microorganisms eventually settle on surfaces, where they accumulate and can be readily resuspended back into the air through human activities such as walking, cleaning, and furniture movement. As a result, surface-deposited fungal communities represent a cumulative assemblage derived from two principal sources: passive settlement of airborne propagules and direct transfer through human contact. Furthermore, direct contact with contaminated surfaces represents a transmission route of comparable importance to respiratory exposure. Therefore, surface sample analysis is essential for characterizing the persistent microbial ecology of indoor environments and assessing risks from both direct contact and secondary aerosolization. By measuring airborne fungal loads through culture-based enumeration and surface community composition through ITS sequencing, this paired design enables assessment of whether ambient environmental conditions that govern airborne fungal concentration also influence the accumulated surface mycobiome, while surface-type-specific analysis further allows direct evaluation of how varying levels of human contact shape community assembly independently of abiotic factors.

Most studies of indoor fungal communities have been conducted in residential facilities or limited building types [7, 8]. In South Korea, investigations of indoor fungi in public facilities have predominantly relied on culture-based methods [9, 10], and the few sequencing-based studies have been limited in scope [11, 12]. Critically, no large-scale study has yet integrated culture-based enumeration (CFU/m^3^) with culture-independent sequencing to comprehensively assess surface mycobiomes across diverse public facility types.

In this study we conducted an extensive sampling across 25 distinct types of public multi-use facilities spanning six major functional categories: transportation hubs, healthcare facilities, educational and childcare institutions, recreational venues, commercial spaces, and specialized controlled environments throughout South Korea. Over a seven-month period (January, March, May, July, August, September, and November 2022), we collected 327 surface samples from six different surface types characterized by varying levels and patterns of human interaction: handles, tables, chairs, walls, pillars, and floors. We applied an integrated approach combining culture-based enumeration (CFU/m^3^ measurements) with culture-independent ITS region sequencing, enabling comprehensive assessment of both fungal loads and taxonomic composition in the public facilities.

## Materials and Methods

### Study Sites and Sample Collections

A total of 327 surface samples and 242 air samples were collected from 25 public facilities of South Korea in January, March, May, July, August, September, and November 2022. To account for environmental variables, atmospheric temperature and relative humidity were recorded at each sampling location at the time of collection. Detailed site information and metadata are summarized in Table S1.

Airborne fungal sampling was conducted according to the Korean Indoor Air Quality Test Methods using a MAS-100eco air sampler at a flow rate of 100 L/min. To ensure statistical reliability, all air samples were collected in triplicate at each accessible location. In cases where multiple surface sampling points shared the same immediate atmospheric environment or where site-specific constraints limited individual measurements, the data were consolidated into a single representative value for that zone. This rigorous filtering process resulted in 242 independent air quality data points, ensuring the statistical independence of the samples used for further analysis. The collected airborne fungi were cultured on Potato Dextrose Agar (PDA) and incubated at 25±1°C for 4–5 days, after which colony-forming units per cubic meter (CFU/m^3^) were calculated using the Feller colony count conversion table.

Surface samples were collected in duplicate from frequently exposed to human activity, such as floors, walls, pillars, tables, chairs, and handles. Swabbing was performed for three minutes using sterile Isohelix Buccal Mini Swabs (Isohelix, Cat.: MS-02, UK), which were then placed into barcoded 2D Matrix V-Bottom tubes (Thermo Fisher Scientific, Cat.: 3741-WP1D-BR/1.0 ml, USA) pre-filled with 400 μl of DNA/RNA shield solution (Zymo Research, Cat.: R1100, USA). All samples were immediately stored at -80°C freezer until DNA extraction. All collection and preservation were followed by the standard operating protocols established by the MetaSUB International Consortium [13].

### DNA Extraction and ITS Sequencing

Total genomic DNA was extracted from swab and pellet using the DNeasy^®^ PowerSoil^®^ Kit (Qiagen Inc., USA), according to the manufacturer's instructions. Samples were homogenized using the Tissue Lyser (Germany) at 18 frequencies for five minutes. After performing quality control (QC), extracted gDNA was subjected to library preparation for analysis by the Illumina MiSeq platform (Illumina, USA). Library preparation followed the Illumina 16S Metagenomic Sequencing Library Preparation protocol (Part # 15044223 Rev. B), adapted for the ITS2 region. The target region ITS2 was amplified with universal primers ITS3 (5'-GCATCGATGAAGAACGCAGC-3') and ITS4 (5'-TCCTCCGCTTATTGATATGC-3'). Illumina overhang adapter sequences were appended to the 5' end of each locus-specific primer, and heterogeneity spacers (0–3 bp) were incorporated between the adapter and locus-specific sequences to increase sequence diversity in the low-diversity amplicon library. The first amplicon PCR was performed using 2× KAPA HiFi HotStart ReadyMix under the following conditions: 96°C for 3 min; 30 cycles of 96°C for 30 s, 55°C for 30 s, and 72°C for 30 s; and 72°C for 5 min. Amplicon PCR products were purified using AMPure XP beads (Beckman Coulter). A second index PCR was performed to attach dual indices and Illumina sequencing adapters using the Nextera XT Index Kit (Illumina) with 2× KAPA HiFi HotStart ReadyMix under the following conditions: 95°C for 3 min; 8 cycles of 95°C for 30 s, 55°C for 30 s, and 72°C for 30 s; and 72°C for 5 min. Indexed libraries were purified with AMPure XP beads, quantified, normalized, and pooled at equimolar concentrations. Sequencing was performed on the Illumina MiSeq platform using paired-end reads.

### Fungi Taxonomic Classification

Initial data quality inspection was performed with FastQC. In the pre-processing step, the primer was removed, and demultiplexing and quality filtering (Phred score ≥20) were applied. A standard denoising pipeline was used to obtain the amplicon sequence variants (ASVs) using the DADA2 plug-in in Quantitative Insights into Microbial Ecology (QIIME) 2, after which the ASVs abundance table was constructed. The obtained ASVs were annotated using NCBI and UNITE databases. After filtering the generated ASVs table using the Biological Observation Matrix (BIOM) format, the resulting sequences were clustered into AVSs based on a similarity threshold ≥97% using Python Nearest Alignment Space Termination (PyNAST). Sequencing reads are deposited in the NCBI Sequence Read Archive (SRA) under BioProject PRJNA1184969.

### Data Visualization and Statistical Analysis

All statistical analyses and data visualization were performed using R software [14]. The significance of difference in community alpha diversity between groups was calculated using Wilcoxon signed rank test, preceded by the Shapiro-Wilk test for normality assessment. The community beta diversity was depicted using non-metric multidimensional scaling (NMDS) plot employing Bray-Curtis dissimilarity distances using the ‘phyloseq’ R package [15]. The analysis of similarity (ANOSIM) was carried out using the R package ‘vegan’ based function ‘anosim’ to compute the significant difference between clusters represented in the NMDS plot [16]. The distances between clusters in the NMDS plots were expressed by computing the average distance between centroids of each group. To account for unequal sample sizes across individual surface types, surfaces were further grouped into high-contact (chairs, tables, and handles) and low-contact (floors, walls, and pillars) categories for statistical comparisons. Liner discriminant analysis effect size (LEfSe) was conducted to identify indicator taxa across surface types and contact-level groups using R package ‘microbiomeMarker’ [17] with data CPM normalization, LDA cutoff value fixed at 3.5, Kruskal-Wallis test cutoff and Wilcoxon signed rank test cutoff at 0.05. To validate LEfSe findings with a compositionally explicit method, differential abundance analysis was additionally performed using ANCOM-BC2 [18], which estimates bias-corrected log-fold changes by accounting for sample-specific sequencing biases inherent in amplicon data. ANCOM-BC2 was applied at both the contact-level (high vs. low) and individual surface-type levels with pairwise comparisons. Taxa with a prevalence below 10% were excluded prior to analysis. Statistical significance was determined at a Benjamini–Hochberg adjusted q-value < 0.05. Two unsupervised co-occurrence networks were inferred from samples classified by the median *Malassezia* relative abundance (2.73%) into low-*Malassezia* (≤ median) and high-*Malassezia* (> median) groups using the Sparse Correlations for Compositional data (SparCC) algorithm using ‘NetCoMi’ and ‘SpiecEasi’ package in R [19, 20]. This algorithm calculates the correlations between microbial genera, considering the sparsity and inherent compositional nature of microbial relative abundance data.

## Results and Discussion

### Comparison of Fungal Communities according to Environmental Factors

To comprehensively evaluate the ecological drivers of indoor fungal communities, we conducted an extensive sampling campaign across 58 public multi-use facilities representing 25 distinct facility types throughout South Korea. Over a seven-month period spanning winter to fall (January, March, May, July, August, September, and November), we collected both air and surface samples from various indoor locations (Table S1).

This dataset represents one of the most comprehensive indoor mycobiome surveys conducted to date, encompassing highly diverse facility types that can be functionally categorized as follows: (1) transportation hubs (subway stations, subway cars, train stations, trains, bus terminals, airports, and port facilities), (2) healthcare facilities (medical facilities, postpartum care centers, and elderly nursing facilities), (3) educational and childcare institutions (libraries, daycare centers, academies, and indoor children's playgrounds), (4) recreational and entertainment venues (sports facilities, movie theaters, PC rooms, museums, exhibition facilities, and indoor performance halls), (5) commercial spaces (large stores and underground stores), and (6) specialized controlled environments (public baths, funeral halls, and indoor parking lots). This extensive facility coverage enabled robust assessment of how different functional contexts shape indoor mycobiomes across the built environment spectrum.

Across all facilities, the recorded temperature averaged 22.1 ± 4.4°C (range: 5.8–28.7°C) and relative humidity averaged 41.0 ± 15.3% (range: 5.4–80.1%) (Table S1). While most indoor facilities maintained relatively stable climatic conditions, certain facility types exhibited substantial microclimatic variation due to their structural characteristics or operational patterns. Facilities with semi-outdoor characteristics or intermittent operation—particularly indoor parking lots and sports facilities—showed pronounced seasonal temperature fluctuations. Indoor parking lots, with direct external connectivity through vehicle entry points, displayed the widest seasonal temperature range (5.8–28.7°C, sd = 7.9°C). Similarly, sports facilities exhibited high variability (sd = 7.5°C), likely reflecting intermittent heating during non-operational hours, which allows indoor temperatures to track seasonal outdoor conditions.

In contrast, the majority of facilities with continuous operation and inherent thermal characteristics maintained stable, elevated conditions. Public baths consistently recorded high temperature (24.7 ± 3.6°C) and humidity (53.7 ± 14.3%) as an inherent consequence of their operational characteristics—continuous hot water supply, steam generation from saunas, and persistent moisture from bathing areas. Postpartum care centers, which require strict climate control to ensure thermal comfort for newborns and recovering mothers, maintained warm conditions (23.8 ± 3.4°C; 42.1 ± 15.2%). Conversely, libraries exhibited exceptionally low humidity (32.6 ± 14.6%; minimum: 5.4%) due to active dehumidification systems designed to prevent moisture-induced degradation of paper-based materials.

**Fig. 1:**
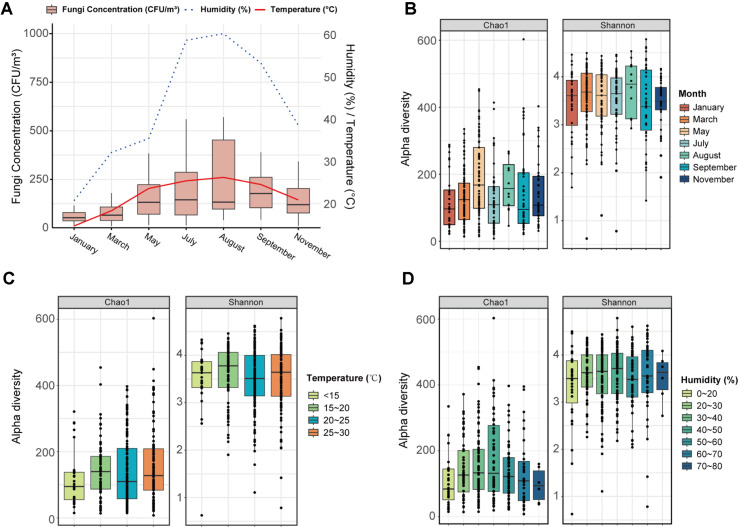
Environmental dynamics of fungal biomass and community diversity in public facilities. (**A**) Seasonal fluctuations in airborne fungal concentration (CFU/m^3^, box plots) in relation to ambient temperature (°C, red line) and relative humidity (%, dotted line). (**B**) Comparison of fungal alpha diversity (Chao1 richness and Shannon diversity indices) stratified by sampling month. Fungal alpha diversity patterns along (**C**) temperature and (**D**) relative humidity gradients.

Beyond these facility-specific microclimatic differences, monthly monitoring revealed a clear seasonal trend in airborne fungal concentrations (CFU/m^3^) (Fig. 1A). Spearman correlations confirmed significant positive associations between CFU and both temperature (R = 0.31, *p*-value < 0.05) and humidity (R = 0.38, *p*-value < 0.05) (Fig. S1A and 1B), consistent with established principles that elevated temperature and moisture promote fungal growth and sporulation [21, 22].

To characterize the stable taxonomic composition and ecological structure of indoor fungal communities, we performed high-throughput sequencing of the ITS region on surface samples. This approach captures a cumulative microbial reservoir, bypassing the high temporal variability inherent in air-sampling while complementing the viability data obtained through air-derived CFU measurements. Sequencing yielded 27 million quality-filtered reads that were clustered into 31,721 amplicon sequence variants (ASVs), demonstrating substantial fungal diversity across indoor environments. In contrast to the clear seasonal pattern observed in fungal quantity (CFU/m^3^), the ecological diversity of the fungal communities remained relatively stable. Analysis of alpha diversity across sampling months showed that while species richness (Chao1) in May exhibited a statistically higher value (*p*-value < 0.05) (Fig. 1B), the Shannon index—which accounts for both richness and evenness—showed no significant differences across months, temperature ranges, or humidity levels (Fig. 1C and 1D, Fig. S1A—1C). The transient increase in Chao1 during May can be attributed to the influx of rare outdoor-associated taxa such as *Physcia*, *Cephaloascus*, and *Monosporascus* [23, 24]. However, these unique genera were present in low relative abundances and did not significantly alter the overall community structure.

Beta diversity analysis corroborated this compositional stability (Fig. S2). Although sampling month showed statistical significance (ANOSIM R = 0.218, *p* < 0.001), the modest R-value indicates substantial overlap between groups, suggesting that seasonal shifts do not fundamentally restructure the core indoor mycobiome. Temperature had no significant effect (R = 0.027, *p* > 0.05), and humidity showed only marginal influence (R = 0.134, *p* < 0.05) (Fig. S2A–2C).

**Fig. 2:**
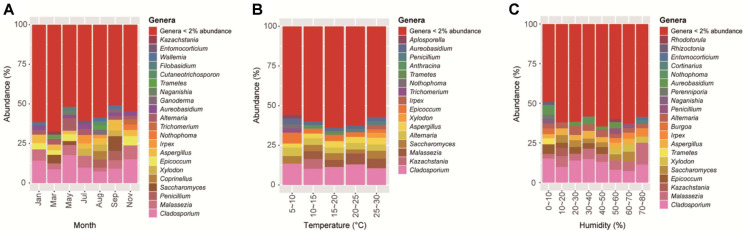
Taxonomic distribution of indoor fungal communities according to environmental variables. Relative abundance of dominant fungal taxa (>2% abundance) at the species level, grouped by (A) month, (B) temperature, and (C) relative humidity.

This stability reflects dominance by a few resilient taxa that maintained high abundances regardless of environmental conditions (Fig. 2A–2C). The most prevalent environmental genera—*Cladosporium*, *Penicillium*, *Aspergillus*, and *Alternaria*—are well-documented cosmopolitan fungi commonly found in indoor air worldwide [25-27]. Additionally, the human-associated genus *Malassezia*, a ubiquitous member of the human skin mycobiota [28, 29], consistently appeared at high relative abundances. The consistent dominance of these taxa maintains low evenness and stable Shannon diversity despite fluctuations in rare species.

Taken together, these findings suggest that while higher temperature and humidity significantly enhance fungal biomass in public facilities, they exert minimal influence on community composition. The persistence of a stable core mycobiome across 25 facility types and substantial climatic variation [30-32] suggests that environmental parameters alone cannot fully explain the ecological structure of indoor mycobiomes. Rather, localized factors—such as the nature of indoor surfaces and the intensity of human contact—may play a more decisive role in defining microbial niche differentiation on specific indoor objects.

### Differences in Microbial Communities on the Objects Subjected to Human Contact

The indoor environment is not a monolithic space, but a mosaic of distinct microbial niches shaped by physical characteristics and human behavioral patterns. Based on the ubiquitous detection of the human-associated genus *Malassezia* in our indoor samples, we hypothesized that the fungal composition in public facilities is significantly driven by the intensity of human contact. To test this, we analyzed the fungal microbiomes of 90 samples categorized into six distinct surface types: chairs (n = 38), floors (n = 28), handles (n = 2), pillars (n = 9), tables (n = 9), and walls (n = 4).

**Fig. 3:**
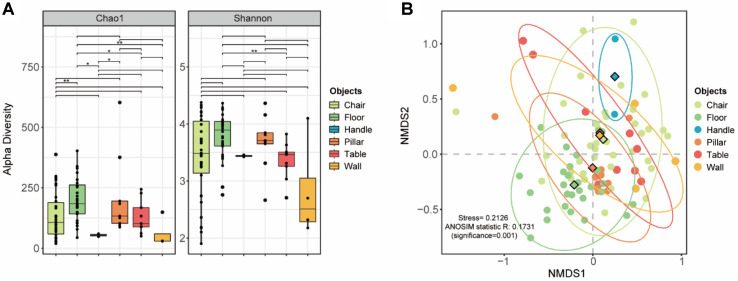
Fungal diversity patterns across indoor surface types. (**A**) Comparison of fungal alpha diversity (Chao1 richness and Shannon diversity) among six surface types. Statistically significant differences are indicated by asterisks (*p < 0.05, **p < 0.01, and ***p < 0.001). (**B**) NMDS ordination based on Bray-Curtis dissimilarity showing fungal community clustering by surface type. Ellipses represent 95% confidence intervals, and diamond symbols indicate group centroids.

Alpha diversity analysis revealed that species richness was significantly influenced by the nature of the surface (Fig. 3A). Floor samples exhibited the highest Chao1 richness, functioning as a “microbial sink” that accumulates a vast array of fungi from both outdoor footwear and the gravity-driven settling [33]. This reduced diversity can be attributed to the synergistic effects of frequent sanitation protocols and the structural nature of these objects—specifically their vertical orientation or limited contact area—which prevents the long-term accumulation of settling bioaerosols. Consequently, the fungal communities on these surfaces are largely restricted to taxa intermittently transferred through direct human contact, limiting the recruitment of diverse environmental species. Despite these differences in richness, Shannon diversity indices remained comparable across all surface types. This trend aligns with our observations in Section 3.1, where the widespread distribution of dominant genera—*Cladosporium*, *Aspergillus*, and *Malassezia*—maintains a consistent level of community evenness regardless of the specific surface type [34].

Beta-diversity analysis provided deeper insights into the "microbial sharedness" between different surfaces. While NMDS ordination showed a degree of overlap (ANOSIM R = 0.173, *p*-value = 0.001), pairwise centroid distance analysis effectively quantified the compositional divergence (Fig. 3B, Table S2). The most significant community dissimilarity was observed between handles and floors (distance = 1.083), representing the two extremes of microbial sources: active human transfer versus passive environmental deposition. Conversely, very small distances were observed between tables and walls (0.022) and chairs and walls (0.053), suggest that surfaces not subject to constant aggressive friction (like handles) share a common fungal profile derived from the settling of the general indoor bioaerosol pool.

**Fig. 4:**
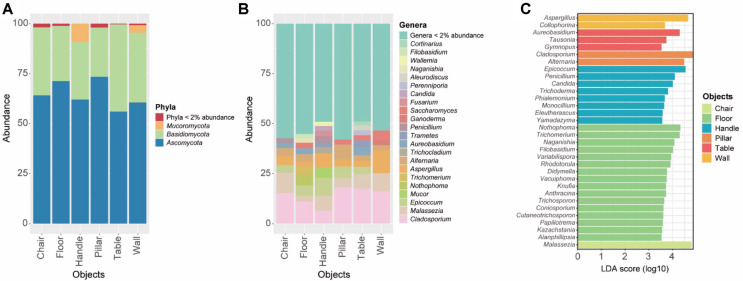
Fungal community composition and indicator taxa across surface types. (**A**) Phylum-level composition of fungal communities by surface type. (**B**) Genus-level distribution of dominant taxa (>2% abundance) across surface types. (**C**) LEfSe analysis identifying indicator taxa for individual surface types (LDA score ≥ 3.5, Kruskal–Wallis and Wilcoxon *p* < 0.05.

The taxonomic composition revealed a clear shift in dominant taxa according to surface characteristics (Fig. 4A and 4B). While Ascomycota (56.0–73.4%) and Basidiomycota (24.6–43.6%) were ubiquitous across all surfaces, genus-level distributions varied significantly. *Malassezia* was notably more abundant on high-contact surfaces such as chairs, handles, and tables compared to low-contact surfaces such as pillars and floors. Conversely, floor samples were characterized by environmentally derived genera such as *Nothophoma*, *Trichomerium*, *Naganishia*, and *Filobasidium*, which are typically associated with soil and decaying organic matter [35-38].

**Fig. 5:**
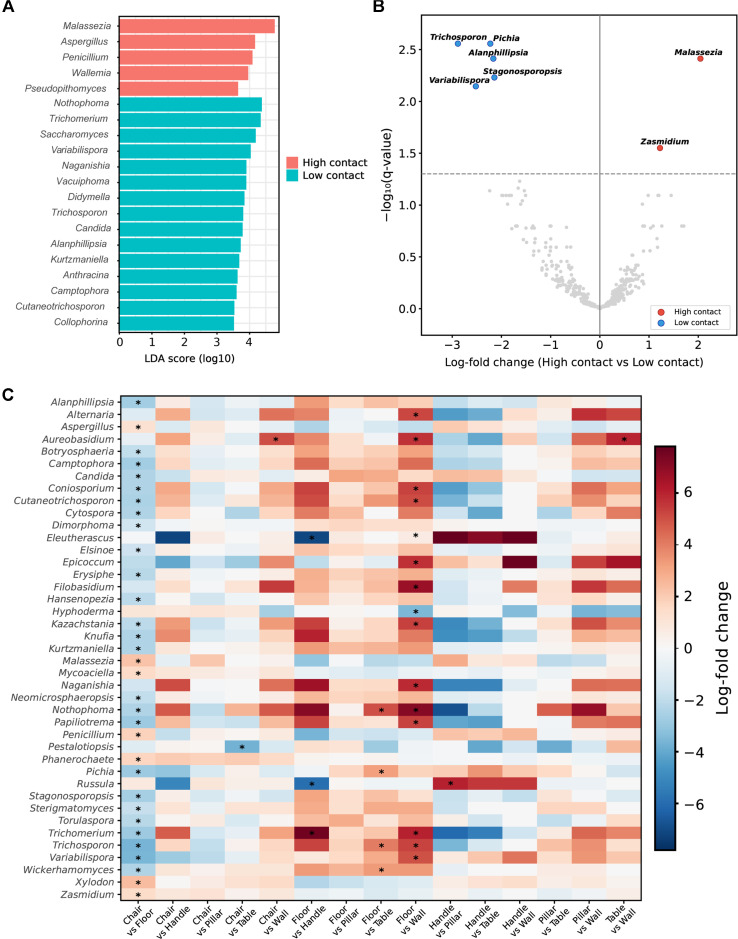
Indicator taxa associated with human contact levels. (**A**) LEfSe analysis comparing high-contact (handles, chairs, and tables; n = 49) and lowcontact (floors, walls, and pillars; n = 41) surface groups. (**B**) Volcano plot of bias-corrected log-fold changes between high-contact (n = 49) and low-contact (n = 41) surface groups. Red and blue points indicate significantly enriched genera (*q* < 0.05, Benjamini–Hochberg correction) in high-contact and low-contact groups, respectively. The dashed line indicates *q* = 0.05. (**C**) Heatmap of bias-corrected log-fold changes across all 15 pairwise surface type comparisons for genera significant (*q* < 0.05) in at least one comparison. Asterisks denote *q* < 0.05.

LEfSe analysis further identified robust indicator taxa that defined the ecological boundaries of these surface niches (Fig. 4C). Soil-associated and phylloplane fungi, such as *Nothophoma*, *Trichomerium* and *Naganishia*, served as significant markers for floor samples, reinforcing the role of floors as a reservoir for outdoor-derived dust and particulates [33, 39]. In contrast, *Malassezia* was identified as a key indicator for chairs, reflecting the extensive transfer of skin mycobiota during prolonged body contact [28]. LEfSe also identified *Fusarium* and *Candida* as indicators of handle surfaces. These genera include clinically significant opportunistic pathogens; *Candida* species are the most common cause of opportunistic mycoses and are frequent inhabitants of human skin and mucous membranes [28, 40], while *Fusarium* species are increasingly recognized as emerging pathogens capable of causing severe invasive infections in immunocompromised individuals [41]. However, given the very limited sample size of handles (n = 2), these associations should be interpreted with caution. To avoid potential bias from sample size imbalance, surfaces were further grouped into high-contact (chairs, tables, and handles; n = 49) and low-contact (floors, walls, and pillars; n = 41) categories for robust statistical comparisons. At the contact-level grouping, LEfSe identified *Malassezia*, *Aspergillus*, and *Penicillium* as indicators of high-contact surfaces, while *Nothophoma*, and *Trichomerium* were indicators of low-contact surfaces (Fig. 5A).

To validate these exploratory LEfSe findings with a compositionally aware method, we performed ANCOM-BC2 analysis comparing high-contact versus low-contact surface groups as well as pairwise comparisons among individual surface types (Fig. 5B and Table S3). At the contact level, ANCOM-BC2 tested 429 genera (prevalence ≥ 10%) and identified seven significantly differentially abundant taxa (*q* < 0.05). *Malassezia* was confirmed as the strongest indicator of high-contact surfaces (log-fold change = +2.04, *q* = 0.004), accompanied by *Zasmidium* (lfc = +1.22, *q* = 0.028) (Fig. 5B). Five genera were significantly enriched on low-contact surfaces: *Trichosporon* (lfc = −2.88, *q* = 0.003), *Variabilispora* (lfc = −2.52, *q* = 0.007), *Pichia* (lfc = −2.23, *q* = 0.003), *Alanphillipsia* (lfc = −2.16, *q* = 0.004), and *Stagonosporopsis* (lfc = −2.14, *q* = 0.006). Pairwise surface-type comparisons further corroborated these patterns, identifying 41 differentially abundant genera across 15 comparisons (Fig. 5C and Table S4). The chair-versus-floor comparison yielded the most contrasts (32 significant genera), with *Malassezia* confirmed as enriched on chairs (lfc = +2.23, *q* = 0.006) and environmental genera such as *Nothophoma* (lfc = +2.33, *q* = 0.035) and *Trichosporon* (Ifc = +3.65, *q* <0.001) confirmed as a floor indicator, consistent with LEfSe results. Cross-validation between the two methods demonstrated strong directional agreement for core taxa: *Malassezia* was fully validated as the primary high-contact indicator across both approaches, and *Nothophoma* and *Trichomerium* were consistently identified as a floor-associated genus. Notably, at the contact level, neither LEfSe nor ANCOM-BC2 identified *Fusarium* or *Candida* as indicators of high-contact surfaces. These handle-specific associations detected only in the surface-type LEfSe analysis likely reflect the influence of the highly imbalanced sample size rather than a genuine ecological pattern. *Candida* was instead found to be significantly enriched on low-contact surfaces in pairwise comparisons (floor > chair, lfc = +2.35, *q* = 0.048). Unlike *Malassezia*, which is an obligate lipid-dependent skin commensal with limited environmental persistence [42], *Candida* is a metabolically versatile genus found in diverse environmental niches beyond human skin [43], and its enrichment on floors likely reflects mixed inputs from both environmental and human sources. These results demonstrate that the core ecological findings—particularly the enrichment of human-associated *Malassezia* on high-contact surfaces and environmental taxa on low-contact surfaces—are robust to the choice of statistical framework, while highlighting the need for caution in interpreting indicators derived from surface types with limited sample representation.

Collectively, these findings demonstrate that human activities and the frequency of physical contact play a decisive role in defining localized fungal niches within public facilities. The clear differentiation between “human-associated” and “environment-associated” indicator taxa suggests that certain fungal genera can serve as proxies for human activity levels, providing a rationale for evaluating the relative abundance of *Malassezia* as a predictive marker for human contact frequency. This role of *Malassezia* as a biological proxy for human contact is consistent with its ecological specialization as the dominant fungal commensal of human skin, where it thrives on lipid-rich sebaceous sites and is intimately associated with skin homeostasis and disease [42]. Importantly, this association is not unique to the facilities surveyed in the present study. In residential environments, touch frequency has been identified as the strongest determinant of surface mycobiome composition, with *Malassezia* being the most prevalent genus across skin, surface, and air samples [44]. Furthermore, fungal communities on residential surfaces are significantly shaped by surface type and occupant presence, with human-associated fungi enriched on frequently contacted surfaces [45]. These converging observations suggest that the human–*Malassezia* signature on built environment surfaces is driven by the universal process of skin shedding rather than region-specific factors such as climate, building design, or hygiene practices.

### Differential Fungal Interactions in Relation to *Malassezia* Abundance

**Fig. 6:**
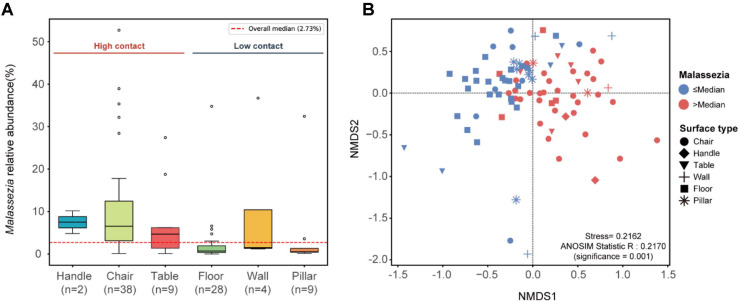
*Malassezia* relative abundance and its influence on community structure. (**A**) Relative abundance (%) of *Malassezia* across six surface types in public facilities. Sample sizes are indicated in parentheses below each surface type. The red dashed line indicates the overall median relative abundance (2.73%) calculated from all 90 surface samples. Surface types are grouped into high-contact (chairs, handles, and tables) and low-contact (floors, pillars, and walls) categories as indicated by brackets. (**B**) Non-metric multidimensional scaling (NMDS) ordination based on Bray–Curtis dissimilarity of 90 surface samples. Points are colored by *Malassezia* relative abundance group: red (>median) and blue (≤median). Shapes represent different surface types. Distinct clustering is confirmed by ANOSIM (R = 0.2170, *p*-value < 0.001, stress = 0.2162).

To explore the ecological implications of human-driven microbial assembly, we compared relative abundance (%) of *Malassezia* across different surface types. Consistent with its role as a proxy for human contact frequency, high-contact surfaces exhibited significantly higher *Malassezia* proportions (median: 5.63%) than low-contact surfaces (median: 1.04%; Mann–Whitney U test, *p* < 0.001) (Fig. 6A). The overall median *Malassezia* relative abundance across all 90 surface samples was 2.73%, and this value was used as the threshold for classifying samples into high- and low-*Malassezia* groups. Consequently, samples from high-contact surfaces primarily exceeded this threshold, whereas those from low-contact environmental surfaces fell below it, indicating a clear bimodal distribution based on the intensity of human interaction.

NMDS ordination revealed that the relative abundance of *Malassezia* significantly partitioned the fungal community into two ecological clusters (ANOSIM R = 0.2170, *p*-value < 0.001, stress = 0.2162) (Fig. 6B). While the R-statistic indicates a moderate degree of overlap, the significant *p*-value confirms that *Malassezia* prevalence is a meaningful determinant of overall community structure. These patterns suggest that human contact does not merely add a few skin-associated taxa but acts as an environmental filter that reshapes the interaction patterns within the indoor mycobiome.

**Fig. 7:**
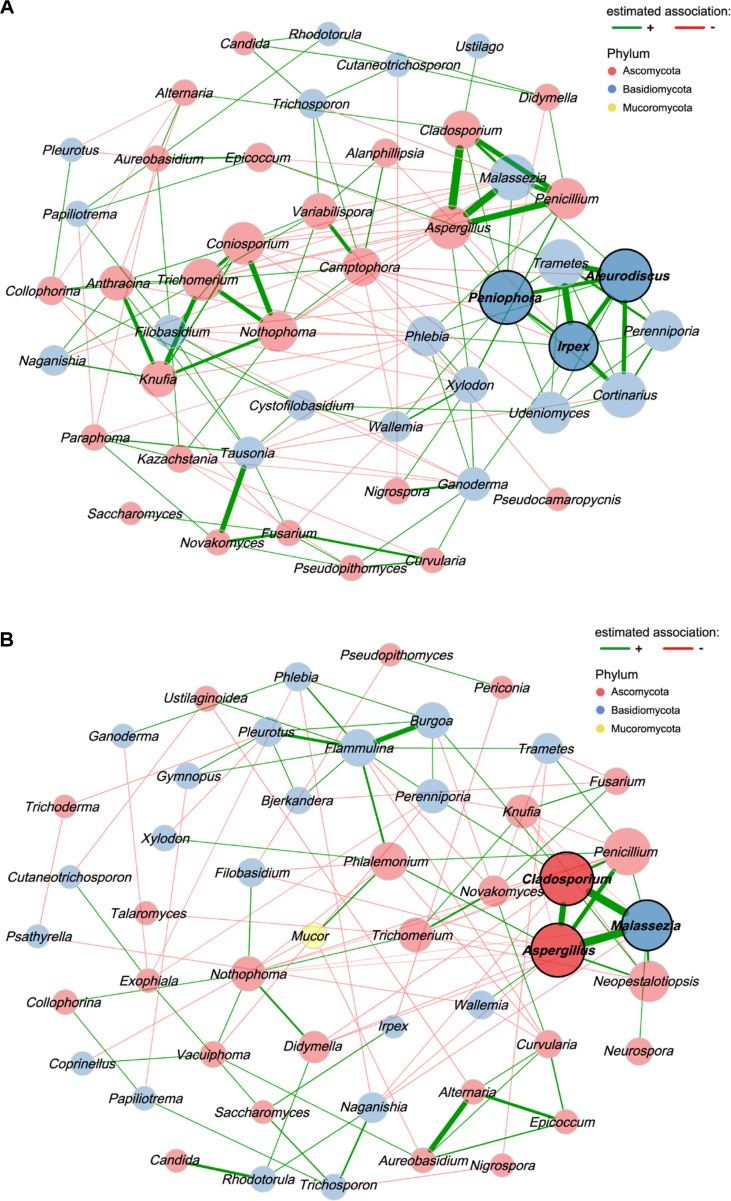
Co-occurrence networks revealing human contact-dependent fungal interactions. SparCC-based co-occurrence networks for samples with (**A**) low-*Malassezia* (≤ median relative abundance, 2.73%) and (**B**) high-*Malassezia* (> median relative abundance, 2.73%). Nodes represent fungal genera, and node size is proportional to eigenvector centrality. Edge colors indicate positive (green) and negative (red) correlations (|corr| > 0.3, *p*-value < 0.05).

To further elucidate these shifts, co-occurrence networks were inferred using the SparCC algorithm, which effectively addresses the sparsity and compositional nature of the fungal dataset. Ecological relevance was maintained by including only significant correlations with |coefficient correlation (= corr)| > 0.3 and *p*-value < 0.05 in the final network construction (Fig. 7). In low-*Malassezia* samples (≤median), the network comprised 50 genera with 107 positive and 50 negative edges (Fig. 7A). This network exhibited an edge density of 0.128 and a modularity index of 0.375, reflecting a structured community with distinct sub-groups. The structural integrity of this environmental network was maintained by wood-decaying and lithic-associated Basidiomycetes, with *Peniophora*, *Aleurodiscus*, and *Irpex* identified as the primary hub nodes based on their high eigenvector centrality. These hubs, along with other high-degree nodes, formed a stable microbial framework reflecting the “environmental sink” nature of surfaces like floors [46]. Notably, even within this environmental network, the human-associated triad of *Malassezia*–*Aspergillus*–*Cladosporium* already exhibited strong positive correlations (*Aspergillus*–*Cladosporium*, |corr| = 0.75; *Malassezia*–*Aspergillus*, |corr| = 0.72; *Malassezia*–*Cladosporium*, |corr| = 0.53), suggesting that human-derived taxa form a coherent ecological module even when their overall abundance is low.

Conversely, the high-*Malassezia* network (>median) showed a slightly lower edge density of 0.092 and a modularity of 0.358, comprising 50 genera with 62 positive and 51 negative edges (Fig. 7B). In this cluster, *Aspergillus*, *Cladosporium*, and *Malassezia* emerged as the primary hub nodes, with *Aspergillus* showing the highest connectivity (degree = 12) and a normalized eigenvector centrality of 1.000, followed by *Cladosporium* (eigenvector = 0.987) and *Malassezia* (eigenvector = 0.897). Notably, exceptionally strong positive correlations were observed between *Malassezia* and *Aspergillus* (|corr| = 0.81), and *Malassezia* and *Cladosporium* (|corr| = 0.81), and *Aspergillus*–*Cladosporium* (|corr| = 0.72). *Penicillium* was also positively associated with this hub triad (*Aspergillus*–*Penicillium*, |corr| = 0.62; *Cladosporium*–*Penicillium*, |corr| = 0.49; eigenvector centrality = 0.796), forming a broader human-associated interaction module. The convergence of these genera as co-occurring hub taxa on high-contact surfaces warrants consideration from a public health perspective. *Aspergillus* encompasses species responsible for a broad spectrum of respiratory diseases, including allergic bronchopulmonary aspergillosis and invasive infections in immunocompromised hosts [25], and *Cladosporium* includes species that have been recovered from clinical specimens of immunocompromised patients [26]. The persistent positive correlation between these two genera across both network states (|corr| = 0.75 and 0.72 in low- and high-*Malassezia* networks, respectively) suggests that they share common ecological strategies for colonizing indoor surfaces. *Candida*, a genus that includes leading agents of opportunistic mycoses [40], showed a positive association with *Rhodotorula* (|corr| = 0.52) in the high-*Malassezia* network, indicating co-occurrence of opportunistic yeast genera on surfaces shaped by human contact [47]. These co-occurrence patterns suggest that high-contact surfaces in public facilities may harbor communities enriched with genera of potential clinical relevance, rather than individual taxa in isolation. The near-equal proportion of positive and negative edges (62 vs. 51) in the high-*Malassezia* network, compared to the positively dominated low-*Malassezia* network (107 vs. 50), indicates a fundamental restructuring of ecological interactions where human-associated hub taxa actively displace environmental genera. The lower modularity (0.358 vs 0.375) compared to the low-contact group suggests a more homogenized community structure, where human-associated shedding acts as a dominant force that integrates diverse taxa into a unified interaction network. This synergistic co-occurrence of genera containing pathogenic and allergenic species on high-contact surfaces suggests that human-mediated transfer creates a unique ecological niche that favors the co-habitation of taxa with potential clinical relevance [48].

This compositional divergence is further evidenced by a strong negative correlation between human-associated hub taxa and environmentally derived fungi. For instance, *Malassezia*, *Aspergillus*, and *Cladosporium* showed significant negative associations with soil- and rock-associated genera such as *Nothophoma* (|corr| = -0.63, -0.59, -0.58, respectively) and *Knufia* (|corr| = -0.56, -0.52, -0.47, respectively). Additional negative correlations were also observed between *Malassezia* and genera like *Naganishia* (|corr| = -0.33), *Didymella* (|corr| = -0.38). Rather than reflecting active biological antagonism, this mutually exclusive distribution pattern likely stems from the contrasting physical environments and human behaviors associated with different surfaces. Frequently touched objects like handles are subject to periodic sanitation and vertical structures that likely limit the passive accumulation of environmental dust, thereby favoring the rapid repopulation of skin-associated taxa through direct contact [3]. In contrast, floor surfaces serve as long-term sinks for gravity-driven settling of environmental particulates, where the sheer volume of environmental ingress outweighs the impact of intermittent human shedding. Overall, these results demonstrate that fungal interaction patterns are bifurcated by the level of human contact, with high-contact surfaces harboring distinct human-associated networks that arise from the combined influence of physical constraints and intensive human interaction.

These findings may also have implications for respiratory health in public spaces. Several genera enriched on high-contact surfaces in this study—including *Aspergillus*, *Cladosporium*, and *Penicillium*—include species that have been associated with allergic sensitization and exacerbation of asthma [49], and *Aspergillus* airway colonization has been frequently reported in patients with COPD, where it is associated with worse clinical outcomes [50]. While inhalation of airborne spores remains the primary route of fungal respiratory exposure, the observation that these genera form densely interconnected hubs on frequently touched surfaces raises the possibility that surface-mediated contact may represent an additional route of exposure. The enrichment of genera containing allergenic and opportunistic species on high-contact surfaces supports the need for integrated air and surface monitoring in public health risk assessments, particularly in facilities serving vulnerable populations.

## Conclusion

This study provides a comprehensive assessment of fungal communities across diverse public facilities, demonstrating that human contact frequency is a primary ecological force shaping the indoor surface mycobiome. While abiotic factors like temperature and humidity significantly influence total fungal biomass (CFU), they exert a marginal effect on community structure and diversity. Instead, the inherent characteristics of indoor surfaces and the intensity of human behavior drive distinct ecological niches within the built environment.

A key finding of this research is the categorization of indoor surfaces into “environmental sinks” and “human-associated hubs”. Surfaces with low human contact, such as floors, function as reservoirs for outdoor-derived fungi, exhibiting high species richness and modular, niche-partitioned networks. In contrast, high-contact surfaces are characterized by lower richness and homogenized fungal communities dominated by human shedding. Crucially, the persistence of these contact-driven patterns across various facility types—irrespective of potential hygiene interventions or cleaning frequencies during the study period—indicates that human-to-surface microbial transfer is a fundamental and resilient ecological process. These patterns were further corroborated by compositionally rigorous differential abundance analysis, confirming the robustness of the core findings across multiple statistical frameworks. Although this study did not include surface-specific absolute quantification methods such as qPCR, the compositional data strongly support a contact-driven restructuring of surface fungal communities; future studies incorporating both compositional and quantitative approaches would further clarify whether human contact is also associated with changes in total fungal biomass.

Furthermore, we established *Malassezia* as a robust biological proxy for human contact levels, as its abundance is strongly associated with the transition from modular environmental networks to centralized, human-integrated networks. The formation of dense interaction hubs among genera of potential clinical relevance, such as *Aspergillus*, *Cladosporium*, and *Candida*, on frequently touched objects highlights the potential for these surfaces to act as focal points for fungal exposure. These insights emphasize the necessity of integrating human behavioral dynamics into future indoor microbiome research, offering a scientific foundation for improved hygiene monitoring, public health risk assessment, and the design of microbial-conscious shared spaces.

## Supplemental Materials

Supplementary data for this paper are available on-line only at http://jmb.or.kr.
